# Association between dietary protein intake and type 2 diabetes varies by dietary pattern

**DOI:** 10.1186/s13098-018-0350-5

**Published:** 2018-06-15

**Authors:** Qiuyi Ke, Chaogang Chen, Fengyi He, Yongxin Ye, Xinxiu Bai, Li Cai, Min Xia

**Affiliations:** 1 Department of Nutrition, School of Public Health, Sun Yat-sen University (Northern Campus), Guangzhou, 510080 Guangdong Province People’s Republic of China; 2 Department of Clinical Nutrition and Endocrinology, Sun Yat-sen Memorial Hospital, Sun Yat-sen University, 107 Yanjiang West Road, Guangzhou, 510120 Guangdong Province People’s Republic of China; 3 Department of Maternal and Child Health, School of Public Health, Sun Yat-sen University (Northern Campus), Guangzhou, 510080 Guangdong Province People’s Republic of China

**Keywords:** Dietary, Protein, Dietary pattern, Type 2 diabetes mellitus

## Abstract

**Background:**

Epidemiological studies have demonstrated that high total protein intake was related to type 2 diabetes mellitus (T2DM) risks. However, few studies considered the impact of dietary pattern.

**Objective:**

We examined the associations between protein intake and T2DM in different dietary patterns.

**Methods:**

We used the demographic and dietary information of adults aged 18–75 years from the China Health and Nutrition Survey (2009), consisting of 4113 women and 4580 men. Dietary data was collected by using 24-h recalls combined with a food inventory for 3 consecutive days. Cluster analysis was used to classify subjects into groups, as determined by major sources of protein. Logistic regression models were used to calculate odds ratios (OR) and 95% confidence interval (95% CI) of T2DM according to the energy-adjusted protein intake.

**Results:**

All participants were divided into three patterns according to the dietary source of protein (legumes and seafood, red meat, refined grains). Overall, plant protein intake was significantly and inversely associated with T2DM. In the subgroup analysis by dietary patterns, extreme quartile of plant protein intake was also inversely related to T2DM in the “legumes and seafood” group [OR = 0.58, 95% CI (0.33–0.96)]. Total protein intake and animal protein intake were positively related to T2DM in the “red meat” group [OR: 3.12 (1.65–5.91) and 3.48 (1.87–6.60), respectively]. However, the association of animal protein intake was reversed in the “refined grains” group [OR = 0.55, 95% CI 0.32–0.89].

**Conclusions:**

The association between protein intake and T2DM varies by dietary pattern. Dietary pattern may be considered into the recommendation of protein intake for diabetes prevention.

## Background

Type 2 diabetes mellitus (T2DM) becomes a major cause of morbidity and mortality globally and contribute considerably to health care costs [1]. The prevalence of T2DM in China has increased substantially from 0.9% in 1980 to 11.6% in 2011 due to the great changes of lifestyles and dietary habits [2]. Therefore, the identification of modifiable risk factors that may contribute to the prevention of T2DM is of essential importance.

Dietary proteins and amino acids are important modulators of glucose homeostasis by promoting insulin resistance and increasing gluconeogenesis [3]. Although high-protein diet has shown beneficial effects on glucose homeostasis in short-term trials [4], emerging evidence suggest that protein actions on T2DM incidence may vary by the amino acid types and food sources. Previous findings from a few long-term epidemiologic studies evaluating food sources of protein reported the conflicting associations of animal and plant protein with risk of T2DM. High total and animal protein intake were associated with a modest elevated risk of T2DM in a large cohort of European adults, but plant protein intake was not associated with T2DM [5, 6]. Higher intake of animal protein such as red and processed meat has been positively associated with risk of T2DM [7], while intake of plant-based sources of protein [8], such as nuts [9], legumes and soy food [10], has been associated with a significantly lower risk of T2DM.

Thus, it is still unclear why the association between different kinds of high-protein food and the risk of T2DM is inconsistent. Furthermore, whether other components in protein-rich foods (e.g., sodium, nitrates, and nitrites in processed red meat), in addition to protein *per se*, may have a critical health effect and account for observed associations.

Patterns of dietary intake reflect an individual’s habitual consumption and would not change for a long time. In practice, each nutrient or food is part of a larger pattern consisting of many nutrients and foods, and thus, characterization of multiple, concurrent dietary exposures have particular relevance to health. It can evaluate individual protein intake in a whole-diet perspective, and make a more practical recommendation for public as dietary guidelines focus on dietary patterns. To date, no studies have considered the association between protein intake and T2DM in different dietary patterns. Therefore, in this study, we extracted and analyzed data from the China Health and Nutrition Survey to determine the association between dietary protein intake and T2DM in different dietary patterns.

## Methods

### Study population

The China Health and Nutrition Survey (CHNS), an ongoing large-scale longitudinal survey initiated in 1989, creates a multilevel method of data collection from all community-dwelling participants and their communities to understand how the wide-ranging set of socioeconomic changes in China affect the health and nutritional status of its population. A multistage random-cluster process was utilized to draw the sample geographically covering 12 provinces in China, which were chosen to generally represent divergence in public resources, health indicators and economic development of all provinces in the country. Eight additional rounds were completed in 1991, 1993, 1997, 2000, 2004, 2006, 2009, and 2011. Details of procedures are described elsewhere [11, 12].

Briefly, to investigate associations between dietary protein and T2DM risk, the cross-sectional data were extracted from the 2009 wave of CHNS, during which fasting blood sample and measurements were collected for the first time. From a total of 9323 eligible adults aged 18 years or older who completed dietary data and biomarker assessment, we excluded 247 participants diagnosed with pregnancy, myocardial infarction or apoplexy, 302 participants with abnormal total energy intake (daily energy intake ≥ 4000 or ≤ 800 kcal/day), and 81 participants with a weight loss diet. Thus, the final analysis consisted of 4113 women and 4580 men.

### Assessment of type 2 diabetes

Blood samples were collected in the morning after overnight fasting via venipuncture by experienced staff, and were frozen at − 86 °C for later laboratory analysis. Plasma glucose and hemoglobin A1c (HbA1c) were measured with standard procedures and strict quality control [11]. T2DM was confirmed according to the diagnostic criterion of HbA1c at or above 6.5%. In contrast to the current diagnostic tests based on point-in-time measures of fasting and postload blood glucose, HbA1c better reflects long-term glycemic exposure and has been demonstrated to be reliable for T2DM diagnosis among Chinese subjects [13].

### Assessment of dietary protein and other nutrients

Before the survey, all field staff, who professionally engaged in nutrition work, were well trained to be acquainted with the containers and food information of region surveyed. 3 consecutive 24-h recalls which were randomly allocated in a week combined with a food inventory over the same three periods to adjust for cooking oil and condiment consumption, were utilized to collect the dietary information. More details about the collection of dietary information can be found elsewhere [14]. The 2002 and 2004 Food Composition Table [15, 16] (Chinese Center For Disease Control And Prevention, Beijing, China) was used to convert food consumption into subjects’ daily intake of nutrients (e.g., protein intake, Energy, fiber, cholesterol). Individual daily intake of each nutrient was adjusted for total energy intake by using the regression residual method [17]. Daily protein intake contributed from each food item was calculated in g/day and grouped into 12 pre-defined food groups (each food group contributing more than 0.5% total daily protein) which were based on similar protein source, nutrient composition, mainly according to the latest 2016 Chinese Dietary Guidelines [18]. The groups included red meat (e.g., pork, beef, and lamb), poultry (e.g., chicken, duck, and goose), dairy (e.g., cow’s milk, yogurt, and milk powder), eggs, seafood (including freshwater fish, e.g., yellow croaker, carp, and shrimp), refined grains (e.g., rice, wheaten food), coarse grains (e.g., oats, maize), tubers, legumes and its products, nuts and seeds, vegetables, and fruits. Then each percentage of total protein intake [protein from specific food group (g/day)/total protein intake (g/day) × 100] was calculated for subsequent cluster analyses.

### Assessment of covariates

Unified trained interviewers administered a detailed questionnaire to collect information including sociodemographic characteristics (e.g., age, gender, education level and annual income), lifestyle factors (e.g., physical activity, smoking status, consumption of tea, coffee and alcohol).

Height (nearest 0.1 cm) and weight (nearest 0.1 kg) were measured in a research clinic affiliated with the academic medical center by trained assessment staff using consolidated tools. Body mass index (BMI) (kg/m^2^) was calculated as weight (kg) divided by height squared (m^2^). Physical activities level (PAL) was administered by addressing the question “PAL involved in work”, whose answer divided into very light (working in a sitting position, office worker, watch repairer, etc.), light (working in standing position, salesperson, teacher, etc.), moderate (student, metal worker, etc.), heavy (farmer, steel worker, etc.) and very heavy (loader, miner, etc.). Furthermore, PAL was quantized into multiples of basal metabolism rate (BMR) according to the basis of Chinese Dietary Reference Intakes [19]: 1.3 × BMR for very light in both sexes, 1.6 and 1.5 × BMR for light, 1.7 and 1.6 × BMR for moderate, 2.1 and 1.9 × BMR for heavy, 2.4 and 2.2 × BMR for very heavy in males and females, respectively. Education level was categorized into low (primary school and lower), middle (middle school and technical or vocational school) and high (college, university and higher). Smoking status was divided into yes (more than once a month or former) and no (never). Consumption of tea, alcohol and coffee were coded as yes (more than once a month) or no (no more than once a month). Annual income was divided into four groups (< 9000; 9000–15,000; > 15,000–25,000; and > 25,000 RMB).

### Statistical analysis

Characteristics of populations were described by proportions for categorical variables, means and standard deviation for normal distribution, and medians and interquartile ranges for skewed distribution of continuous variables. Total, animal, and plant protein intake, with adjustment for total energy intake by the regression residual method [17], were categorized into quartiles respectively.

Dietary patterns were derived by protein-rich food groups, using fast cluster models in cluster package. Firstly, the percentage of total daily protein that was contributed from each food was calculated for each individual. Foods containing protein were grouped into 12 predefined food groups on the basis of nutrient-composition similarities, protein type, or source according to mainly according to the latest 2016 Chinese Dietary Guidelines. Secondly, dietary patterns were derived by protein-rich food group, using fast cluster models in cluster package. The technique applied K-means method of cluster analysis to categorized subjects into mutually exclusive groups by Euclidean distance between each person and each cluster center in an iterative process. We excluded participants whose protein contributions from food groups were 5 standard deviations away from the mean protein contributions and verified each food groups contributing more than 0.5% total daily protein because cluster analysis is sensitive to outliers. Thirdly, we ran predetermined numbers of clusters (2–6 times) to determine the most meaningful interpretation according to dietary feature of Chinese population. The 3-cluster set was chosen because it presented the most meaningfully separated clusters, also including a high F ratio (mean square between clusters/mean-squared error), and each clusters distributed participants well between all clusters (each cluster contained more than 100 subjects). Naming of clusters was determined by the value which represent the highest consumption of one or two food groups compared with other clusters. The methods were previously performed in other studies [20, 21], and discussion (such as attention, background) of cluster methods have been described elsewhere [22, 23].

Logistic regression models were used to calculate crude, adjusted odd ratios (ORs) and 95% confidence interval (CI) for the associations of quartiles of energy-adjusted protein intake, animal protein intake, and plant protein intake with T2DM. P for trend was conducted by taken the median of each energy-adjusted protein intake quartile as continuous variables in the logistic regression models. In multivariate models, model was adjusted for age and sex firstly. In the second model, model was further adjusted for the covariates, such as PAL, smoking status, alcohol consumption, tea consumption, coffee consumption, annual income and education (low, middle, or high). In the third model, the nutritional factors was added to the model, included total energy, carbohydrate to energy ratio from refined grains or tubers, from the other plant sources and energy-adjusted intake of saturated fat, monounsaturated fat, polyunsaturated fat, fiber, cholesterol. In the last model, BMI was additionally considered. Subgroup analysis by dietary protein food patterns was conducted to explore the relation between energy-adjusted protein intake with prevalence of T2DM in mutually exclusive subjects with different dietary preferences. Data was analyzed by R software (version in 3.4.1).

## Result

### Characteristics of study subjects

The characteristics of 8693 participants (4113 women and 4580 men) from the 2009 wave of CHNS were shown in Table 1. Participants were categorized into quartiles of energy-adjusted total protein intake. Only actual daily dietary intake without energy adjustment were presented in the daily nutrient intakes of Table 1, but energy-adjusted nutrients intakes were applied in the following statistical analysis.

**Table 1:** Characteristics and dietary consumption in a cross-sectional study by categories of energy-adjusted total protein intake

	Q1:50.8 g/day (46.3, 53.6)^a^	Q2:59.6 g/day (57.8, 61.5)^a^	Q3:68.0 g/day (65.7, 70.4)^a^	Q4:81.6 g/day (76.3, 89.9)^a^
Demographic and lifestyle factors				
N (cases)	2173 (146)	21,734 (128)	2173 (145)	2174 (142)
BMI (kg/m^2^)	23.0 ± 3.5	23.2 ± 3.5	23.4 ± 3.4	23.6 ± 3.5
PAL (×BMR)	1.70 ± 0.32	1.65 ± 0.30	1.62 ± 0.29	1.60 ± 0.29
Residence area				
Urban (%)	19.8	26.2	36.5	44.4
Gender				
Male (%)	49.1	43.4	46.0	50.6
Education level				
Low (%)	52.7	46.0	39.9	32.3
Middle (%)	44.3	50.1	53.6	60.1
High (%)	3.0	3.9	6.4	7.6
Annual income, RMB				
< 9000	68.5	65.2	53.6	50.7
9000–15,000	16.4	18.9	22.3	21.9
> 15,000–25,000	10.4	10.7	14.9	16.9
> 25,000	4.7	5.2	9.2	10.5
Former or current smoking (%)	33.4	30.2	28.0	33.1
Tea consumption (yes, %)	29.0	29.6	31.7	36.4
Coffee consumption (yes, %)	1.3	1.7	2.4	4.0
Alcohol consumption (yes, %)	20.9	17.3	20.7	22.2
Daily nutrient intakes				
Total energy (kcal/day)^b^	2277.1 ± 658.0	1976.1 ± 582.4	2031.8 ± 595.7	2234.5 ± 618.6
Total protein (g/day)^b^	52.4 ± 15.7	55.5 ± 15.5	65.4 ± 16.0	87.9 ± 22.6
Total protein (energy%)^b^	9.2 ± 1.1	11.3 ± 0.6	13.1 ± 1.0	16.0 ± 2.4
Animal protein (g/day)^b^	10.6 ± 8.8	12.8 ± 8.9	17.7 ± 10.4	29.0 ± 16.4
From red meat (g/day)^b^	7.2 ± 7.5	8.3 ± 7.7	10.5 ± 9.3	18.0 ± 14.5
From poultry (g/day)^b^	0.8 ± 2.3	1.0 ± 2.7	2.1 ± 4.4	4.3 ± 7.0
From dairy (g/day)^b^	0.1 ± 0.8	0.2 ± 1.0	0.5 ± 2.1	0.8 ± 2.8
From egg (g/day)^b^	2.2 ± 2.9	3.0 ± 3.5	3.9 ± 4.0	5.0 ± 5.6
From seafood (g/day)^b^	1.4 ± 2.9	2.0 ± 3.9	3.5 ± 5.3	7.3 ± 9.7
Plant protein (g/day)^b^	37.8 ± 13.5	37.7 ± 13.9	40.3 ± 16.1	47.1 ± 21.7
From grain (g/day)^b^	28.7 ± 11.4	28.0 ± 11.9	28.5 ± 13.7	27.0 ± 13.9
From coarse cereals (g/day)^b^	2.0 ± 3.8	1.7 ± 2.7	1.5 ± 2.8	1.3 ± 2.5
From vegetable (g/day)^b^	4.6 ± 3.0	4.5 ± 3.1	4.7 ± 3.3	5.5 ± 3.8
From fruit (g/day)^b^	0.2 ± 0.5	0.2 ± 0.5	0.2 ± 0.5	0.3 ± 0.6
From legumes (g/day)^b^	2.3 ± 3.8	3.2 ± 4.8	5.2 ± 6.5	11.3 ± 14.3
From nuts and seeds (g/day)^b^	0.3 ± 1.7	0.5 ± 2.0	0.7 ± 3.0	1.0 ± 3.5
From tubers (g/day)^b^	0.4 ± 0.4	0.4 ± 0.4	0.7 ± 0.6	1.9 ± 0.7
Total fat (g/day)^b^	86.3 ± 42.7	66.6 ± 30.8	67.5 ± 30.1	78.0 ± 31.9
Total fat (energy%)^b^	33.7 ± 11.6	30.1 ± 9.8	29.9 ± 9.6	31.4 ± 9.2
Saturated fat (g/day)^b^	10.7 ± 9.0	7.2 ± 5.4	7.2 ± 5.2	8.5 ± 5.6
Monounsaturated fat (g/day)^b^	21.2 ± 15.5	14.3 ± 10.6	14.0 ± 10.0	16.2 ± 11.1
Polyunsaturated fat (g/day)^b^	19.4 ± 17.2	13.7 ± 10.0	13.3 ± 9.5	14.2 ± 9.6
Total carbohydrate (g/day)^b^	324.5 ± 105.9	296.1 ± 98.6	299.2 ± 105.7	305.4 ± 106.0
Total carbohydrate (energy%)^b^	57.7 ± 11.7	60.2 ± 10.3	58.8 ± 10.2	54.5 ± 10.0
Cholesterol (mg/day)^b^	171.1 ± 154.7	206.8 ± 166.9	269.3 ± 175.4	395.1 ± 270.4
Fiber (g/day)^b^	10.5 ± 5.8	10.1 ± 5.0	11.6 ± 7.2	13.9 ± 8.9

Values were presented as mean ± standard deviation, median (IQRs) or proportions

^a^ Estimated intake energy adjusted by the residual method

^b^ Actual daily nutrients intake without energy adjustment

Over the quartiles of energy-adjusted total protein intake, mean dietary intakes of all kinds of animal protein (total animal protein and the protein from red meat, poultry, seafood, dairy and egg), plant protein from nuts and seeds, legumes and cholesterol increased, whereas mean dietary intake of protein from coarse cereals decreased. Participants who consumed more daily protein had high education level, annual income, tea and coffee consumption, proportion of urban residents, BMI and lower level of physical activity.

### Dietary protein food patterns and the association with T2DM

Subjects were categorized into three different dietary protein food patterns, whose name were determined by the highest percentage of intake from one or two food groups. Percentage protein contribution from each specific food group was shown in Table 2. Compared to other groups, the “legumes and seafood” dietary pattern (mean percentage protein contribution from legumes and seafood: 14.3 and 8.7, n = 2984) presented with a relatively higher protein intake from legumes, seafood, nuts and seeds, coarse cereals, fruits, poultry, dairy, and eggs. The “Red meat” and “refined grains” dietary patterns presented with higher protein consumption from red meat (33.1%) and refined grains (63.5%) respectively.

**Table 2:** Average percentage of total protein intake from individual food group across protein food cluster analysis of 8774 men and women from the CHNS study

Food group	Legumes and seafood	Red meat	Refined grains
N (case)	2984 (182)	2569 (174)	3140 (204)
Red meat, %	10.9 ± 7.1	33.1 ± 9.9	7.3 ± 7.6
Poultry, %	4.1 ± 7.5	3.3 ± 6.0	1.3 ± 4.2
Dairy, %	1.0 ± 3.9	0.6 ± 2.3	0.1 ± 1.2
Egg, %	6.7 ± 6.6	4.3 ± 5.2	5.3 ± 6.2
Seafood, %	8.7 ± 10.1	4.7 ± 6.6	1.8 ± 4.4
Refined grains, %	34.2 ± 10.2	33.9 ± 10.2	63.5 ± 11.0
Coarse cereals, %	5.4 ± 9.2	1.9 ± 4.0	4.1 ± 6.0
Vegetables, %	7.8 ± 5.0	8.0 ± 4.6	8.3 ± 5.1
Fruits, %	0.5 ± 1.1	0.4 ± 0.9	0.2 ± 0.7
Legumes, %	14.3 ± 13.6	4.8 ± 6.1	3.9 ± 5.8
Tubers, %	0.2 ± 1.0	0.2 ± 0.8	0.1 ± 0.4
Nuts and seeds, %	1.3 ± 4.2	0.7 ± 2.5	0.5 ± 2.6

A K-means cluster analysis was used to classify participants into mutually exclusive groups

Naming of clusters was determined by the value which represent the highest consumption of one or two food groups compared with other clusters

Percentage of total protein intake across the each food group was used

Mean ± SE

In multivariate-adjusted models, when compared to the T2DM prevalence of the “legumes and seafood” dietary pattern, ORs (95% CI) for T2DM were 1.49 (1.15, 1.97) and 1.45 (1.12, 1.91) in the “red meat” and “refined grains” respectively (Table 3).

**Table 3:** The odds ratios of type 2 diabetes across three dietary patterns

Dietary pattern	HR (%)	OR_1_	OR_2_	OR_3_	OR_4_
Legumes and seafood	6.1	1	1	1	1
Red meat	6.8	1.33 (1.05, 1.66)	1.38 (1.09, 1.75)	1.43 (1.11, 1.85)	1.49 (1.15, 1.97)
Refined grains	6.5	1.23 (0.99, 1.55)	1.39 (1.10, 1.78)	1.40 (1.11, 1.80)	1.45 (1.12, 1.91)

Model 1: adjusted age (continuous), sex (male, female); Model 2: Model 1 + PAL (continuous), smoking status (yes, no), alcohol consumption (yes, no), coffee consumption (yes, no), tea consumption (yes, no), annual income (< 9000; 9000–15,000; > 15,000–25,000; > 25,000) and education (low, middle, high); Model 3: Model 2 + total energy intake (continuous), carbohydrate to energy ratio from refined grains or tubers, from the other plant sources (continuous), energy-adjusted intake (continuous) of total protein, SFA, PUFA, MUFA, fiber, and cholesterol; Model 4: Model 3 + BMI (continuous)

### Subgroup analysis of the association between protein intake and T2DM by dietary protein food patterns

After adjustment for covariates, the OR for T2DM over extreme quartiles (highest vs. lowest) of energy-adjusted total protein intake was 3.12 [95% CI 1.65–5.91; *P* for trend < 0.001] in the “red meat” group (Table 4), and there is no significant association between total protein intake and T2DM in the total population [OR: 1.23 (0.89, 1.69)].

**Table 4:** The odds ratios of type 2 diabetes across quartiles of energy-adjusted total protein intake by dietary patterns

	Energy-adjusted total protein intake quintiles, OR (95% CI)				P for trend
	Q1	Q2	Q3	Q4	
Total protein					
Overall	1	0.89 (0.66, 1.20)	1.09 (0.80, 1.46)	1.23 (0.89, 1.69)	0.117
Legumes and seafood	1	0.65 (0.37, 1.10)	0.97 (0.58, 1.61)	0.78 (0.45, 1.36)	0.892
Red meat	1	1.74 (0.97, 3.27)	2.80 (1.57, 5.01)	3.12 (1.65, 5.91)	< 0.001
Refined grains	1	0.93 (0.58, 1.51)	1.02 (0.62, 1.69)	1.06 (0.63, 1.81)	0.298
Animal protein					
Overall	1	0.76 (0.56, 1.02)	0.81 (0.59, 1.12)	1.32 (0.93, 1.89)	0.104
Legumes and seafood	1	0.80 (0.47, 1.37)	0.85 (0.50, 1.51)	1.03 (0.54, 1.77)	0.770
Red meat	1	2.42 (1.38, 4.33)	2.87 (1.57 ,5.34)	3.48 (1.87, 6.60)	< 0.001
Refined grains	1	0.70 (0.45, 1.10)	0.64 (0.40, 1.03)	0.55 (0.32, 0.89)	< 0.05
Plant protein					
Overall	1	0.86 (0.65, 1.14)	0.75 (0.57, 1.02)	0.72 (0.51, 0.95)	< 0.05
Legumes and seafood	1	0.77 (0.48, 1.24)	0.64 (0.40, 1.01)	0.58 (0.33, 0.96)	< 0.05
Red meat	1	1.48 (0.87, 2.63)	1.28 (0.74, 2.27)	1.20 (0.628, 2.21)	0.205
Refined grains	1	0.88 (0.54, 1.42)	1.06 (0.67, 1.69)	1.09 (0.67, 1.81)	0.289

^a^ Adjusted age (continuous), gender (male, female), PAL (continuous), smoking status (yes, no), alcohol consumption (yes, no), coffee consumption (yes, no), tea consumption (yes, no), annual income (< 9000; 9000–15,000; > 15,000—25,000; > 25,000), education (low, middle, high), total energy intake (continuous), carbohydrate to energy ratio from refined grains or tubers, from the other plant sources (continuous), energy-adjusted intake (continuous) of SFA, PUFA, MUFA, fiber, and cholesterol, BMI (continuous)

In the total population, animal protein intake demonstrated a non-significant positive relation with T2DM [OR: 1.32 (0.93, 1.89)]. In the subgroup analyses, there was a positive association between animal protein intake and T2DM in the “red meat” group [OR: 3.48 (1.87, 6.60)]. However, the association between animal protein intake and T2DM was significantly inversed in the “refined grains” group [OR: 0.55 (0.32, 0.89)].

Overall, plant protein intake was significantly inversed related to T2DM after adjustment for all covariates [OR: 0.72 (0.51, 0.95)]. Moreover, plant protein intake was negatively related to T2DM [OR: 0.58 (0.33, 0.96)] in the “legumes and seafood” subgroup. This association was not significant in the “red meat” and “refined grains” dietary patterns. Sensitivity analyses excluding subjects with previously diagnosed type 2 diabetes was repeated in the statistical analysis and similar results were obtained (data not shown). Additional analyses were also conducted to assess the associations of quartiles of energy-adjusted protein intake from different animal or plant sources with T2DM. Protein intake from red meat [OR: 2.34 (1.35, 4.12)], or refined grains [OR: 2.15 (1.37, 3.41)] was positively related with T2DM risks. In contrast, protein intake from legumes [OR: 0.57 (0.32, 0.95)], or vegetables [OR: 0.66 (0.37, 0.94)] was negatively related with T2DM risks. There was no significant association between protein intake from poultry, dairy, eggs, seafood, coarse cereals, tubers, nuts and seeds, or fruits (data not shown).

## Discussion

In this study, higher intake of vegetable protein was negatively associated with T2DM risk in Chinese population. Furthermore, after categorizing subjects into different dietary protein food patterns, the inverse association between plant protein intake and T2DM risk remained in the “legumes and seafood” dietary pattern. The positive relation between total protein and animal protein with T2DM risk were significant and independent in the “red meat” dietary pattern, whereas in the “refined grains” dietary patterns, animal protein intake was associated with a lower T2DM risk. These finding suggested that considering protein intake from a whole-diet perspective of dietary pattern is necessary for T2DM prevention.

Our findings of positive association between total and animal protein intake with T2DM risk in the “red meat” dietary pattern was consistent with previous studies reported in northeastern Chinese, American, and European populations [5, 6, 8, 24]. The quantity and composition of protein intake in the “red meat” dietary pattern was similar with these studies. Participants have consumed a relatively high protein intake (even more than 50% of protein intake from animal protein). As for plant protein intake, our finding demonstrating a modest inverse association between plant protein intake and T2DM, was consistent with the pooled analysis of NHS, NHS II, and HPFS, which reported that whole grains, nuts, peanut butter, and beans were the main sources of plant protein intake [8]. However, most previous individual studies [5, 6, 8, 24] showed no significant association of plant protein intake with T2DM risks. The divergence might occur that plant protein were from different sources across different study populations.

Actually, dietary protein food patterns that last for a long period for a person and hardly change totally, can reflect the divergent sources of plant protein and animal protein [20, 21]. Besides, nowadays dietary guidelines also focus on dietary patterns [25]. Therefore, the question was raised as to whether different relation may be occurred between protein intake with T2DM in various dietary protein food patterns.

Initially, we observed three typical dietary protein food patterns in the 2009 wave of CHNS. People with “legumes and seafood” dietary pattern consumed nearly 30% percentage of animal protein, and only 1/3 of the animal protein was from read meat. This protein food pattern represents the traditional Chinese diet, which grains eaters foremost with high consumption of legumes and vegetables, and moderate use of animal food. It presented the lowest T2DM prevalence, lining with previous observations [26–28] that the dietary patterns rich in legumes, fruits and vegetables had a favorable effect on the prevention of T2DM. On the other hand, the “refined grains” dietary pattern had nearly 65% percentage of protein from refined grains. It was another typical Chinese diet, which consists of a variety of cereal products and tubers, contributing as the primary source of nutrients intake. Previous studies demonstrated this kind of dietary pattern was positively associated with diabetes [14, 28–30]. Not only high intake of refined grains is the pivotal individual risk factors related to Chinese diabetes burden, high intake of red meat also contributes Chinese diabetes burden [29, 31].

Furthermore, our results showed that the relation of protein intake to T2DM varied by dietary protein food patterns. The underlying molecular mechanism of divergent associations between protein intake and T2DM remain unclear, but potentially was related to the other components of the high intake of various protein-rich food sources. Additionally, this discrepancy also could not be ignored because of the differences in amino acid and protein composition. Not all protein sources modulate insulin secretion and insulin sensitivity with equal abilities in healthy and T2DM populations. Because certain dietary proteins, peptides and amino acids can directly affect insulin secretion and insulin sensitivity. For example, some amino acids are believed to interfere with insulin’s ability to increase peripheral glucose uptake in skeletal muscle, or intervene with glucose metabolism via stimulation of insulin and glucagon secretion and by serving as substrates for gluconeogenesis. Furthermore, certain dietary proteins, peptides and amino acids can indirectly influence the intermediate substance of insulin secretion such as glucose-dependent insulin tropic peptide (GIP) and glucagon-like peptide-1 (GLP-1) secretion [32, 33].

For the “legumes and seafood” dietary pattern, higher intake of plant protein reduced T2DM risks, which may be in part due to that increased protein intake was accompanied with more consumption of legumes, nuts and seeds, whole grains, vegetables, and fruits. These foods were proven to be beneficial for preventing T2DM due to high content of fiber, magnesium and vitamin [25, 34]. However, the association still existed after adjustment for these nutrient intakes. Thus, we may be able to propose a possibility that protein *per se* of these food items potentially benefited to decrease T2DM risk. The result was consistent with previous study that replacing meat protein with soy protein altered insulin resistance and blood lipids [35]. The mechanisms underlying the beneficial effect of soy protein might be explained for inhibiting lipogenesis or insulin secretion from pancreatic β cells, which were believed to enhance lipolysis in the adipose and liver to reduce adiposity [36]. Additionally, a clinical trials found that changing dietary protein sources to plant and fish-based sources lowered the plasma branched chain amino acids (BCAAs) concentrations, which have been shown to be positively linked to diabetes risk [37]. Furthermore, insulin-like plant proteins may partly attribute to the relation. In several vitro and in vivo studies, insulin-like plant proteins was isolated from leave, seeds and fruits, which was proven to increase tolerance to orally administered glucose with hypoglycemic activity in chemically induced diabetic mice [38–40].

Contrary to the negative association between plant protein and T2DM in the “legumes and seafood” dietary pattern, the “refined grains” pattern leads an insignificant association. This may due to the different protein-rich food sources. Satija’s study showed that not all plant foods are necessarily beneficial and found varied incidence of T2DM in different plant-based dietary patterns [25]. However, animal protein presented a protective effect in the “refined grains” dietary pattern, in which animal protein was less than 20% and mainly contributed from dairy, seafood, poultry and eggs. A high quality of the protein cluster, which was consisted of an optimal composition, may partly explain the benefit of relatively high animal protein. In most cases, the concentrations of crude protein and amino acid in the cereal grains, especially several cereal grains most available for human consumption, are not sufficient to fulfil crude protein and amino acid requirements for proper growth and development [41]. Wu et al. [42] proposed that an optimal composition of dietary amino acids could result in decreased risks of obesity and T2DM by enhancing the efficiency of amino acid metabolism. Findings from a prospective population-Based Study also reported that an unbalanced amino acid patterns might lead to dysglycemia risk. Thus, appropriate amount animal protein from these food items was recommended to the “refined grains” patterns.

However, higher animal intake increased T2DM risks in the “red meat” dietary pattern. As the main animal protein sources, red meat was reported to increase T2DM risks independent of fat intake [43, 44]. Heme iron, nitrites and advanced glycation end products are thought to mediate the association [7]. In our study, after adjusting estimated iron intake and processed meat intake, the association was attenuated but remained significant. This result was similar with the results of Nurses’ Health Study [8], which suggested that the direct effect of protein from meat cannot be excluded. This association may mainly be due to amino acid composition. Floegel et al. [45] found BCAAs performed a high circulation level after red meat intake. Compared with a meal mainly from vegetable sources, a meal mainly from red meat sources leads an even nearly 100% higher plasma concentrations of BCAAs [46]. Furthermore, Wu et al. found that BCAA contributes to develop insulin resistance in a poor dietary pattern including high fat consumption. The results of Metabolite-profiling studies also reported five branched-chain and aromatic (isoleucine, leucine, valine, tyrosine and phenylalanine) were highly associated with diabetes [47]. It suggested BCAAs activated mammalian target of rapamycin complex 1 (mTORC1), so that caused insulin resistance [48] although relation of amino acid to T2DM risk need to be further elucidated.

Limitations of this study were as follows. Firstly, we cannot establish causal relationship as our study was cross-sectional. However, we have run sensitivity analyses excluding subjects with previously diagnosed type 2 diabetes and found similar results, demonstrating that the possibility of reverse causation which subjects changed their diet following diagnosis was less likely to happen. Large-scale longitudinal study and long-term study are warranted. Secondly, the 3 consecutive 24-h recalls randomly allocated in a week may not reflect the long-term protein intake. However, it was suggested that duplicate 24-h recalls could be used to asses intakes of nutrients, such as protein, carbohydrates, starch, sugar, water, potassium and calcium [49], and the 3 consecutive days were used in many population studies [50, 51]. In this study, we also adjusted the condiments intake according to a food inventory of household. Thirdly, potential residual confounding may exist although we adjusted a number of potential confounders in analyses. Fourthly, there were the disadvantages to cluster analysis including sensitivity to outliers and subjective interpretation of the clusters after the complete statistical model. Thus, we employed the details described in methods to remove the outliers and set standards for outlined decisions of the cluster.

## Conclusions

The results of our study suggested the associations between protein intake and T2DM vary by dietary pattern. Dose of protein intake may interact with dietary patterns. Our study suggested that dietary pattern may be considered into the recommendation of protein intake for diabetes prevention.

## Abbreviations

T2DM: type 2 diabetes mellitus
OR: odds ratios
95% CI: 95% confidence interval
CHNS: China Health and Nutrition Survey
HbA1c: hemoglobin A1c
PAL: physical activities level
BMR: basal metabolism rate
BMI: body mass index
GIP: insulinotropic peptide
GLP-1: glucagon-like peptide-1
BCAAs: branched chain amino acids
mTORC1: mammalian target of rapamycin complex 1
